# A Growth Factor Attenuates HIV-1 Tat and Morphine Induced Damage to Human Neurons: Implication in HIV/AIDS-Drug Abuse Cases

**DOI:** 10.1371/journal.pone.0018116

**Published:** 2011-03-24

**Authors:** Shaily Malik, Hena Khalique, Shilpa Buch, Pankaj Seth

**Affiliations:** 1 Cellular and Molecular Neuroscience, National Brain Research Center, Manesar, Gurgaon, Haryana, India; 2 Department of Pharmacology and Experimental Neuroscience, University of Nebraska Medical Center, Omaha, Nebraska, United States of America; George Mason University, United States of America

## Abstract

The neuropathological abnormalities of human immunodeficiency virus (HIV)-1 patients abusing illicit drugs suggest extensive interactions between the two agents, thereby leading to increased rate of progression to neurodegeneration. The role of HIV-1 transactivating protein, Tat has been elucidated in mediating neuronal damage via apoptosis, a hallmark of HIV-associated dementia (HAD), however the underlying mechanisms involved in enhanced neurodegeneration by illicit drugs remain elusive. In this study, we demonstrated that morphine enhances HIV-Tat induced toxicity in human neurons and neuroblastoma cells. Enhanced toxicity by Tat and morphine was accompanied by increased numbers of TUNEL positive apoptotic neurons, elevated caspase-3 levels and decreased ratio of anti- and pro-apoptotic proteins, Bcl2/Bax. Tat and morphine together elicited high levels of reactive oxygen species that were NADPH dependent. Significant alterations in mitochondrial membrane homeostasis were also observed with co-exposure of these agents. Extensive studies of mitogen activated protein kinase (MAPK) signaling pathways revealed the involvement of c-Jun N-terminal kinase (JNK) and extracellular signal-regulated kinase-1/2 (ERK1/2) pathways in enhanced toxicity of Tat and morphine. In addition to this, we found that pre-treatment of cells with platelet derived growth factor (PDGF-BB) protected neurons from HIV-Tat and morphine induced damage. PDGF-BB alleviated ROS production, maintained mitochondrial membrane potential, decreased caspase-3 activation and hence protected the cells from undergoing apoptosis. PDGF-BB mediated protection against Tat and morphine involved the phosphatidylinositol–3 kinase (PI3K) pathway, as specific inhibitor of PI3K abrogated the protection conferred by PDGF-BB. This study demonstrates the mechanism of enhanced toxicity in human neurons subjected to co-exposure of HIV protein Tat and morphine, thus implying its importance in HIV positive drug abusers, where damage to the brain is reported to be more severe than non-drug abusers. We have also showed for the first time that PDGF-BB can protect against simultaneous exposure of Tat and morphine, strengthening its role as a neuroprotective agent that could be considered for therapeutic intervention.

## Introduction

Neurological dysfunction in human immunodeficiency virus (HIV)/acquired immunodeficiency syndrome (AIDS) patients results either due to the presence of the virus in the nervous system itself or indirectly by immune cell depletion and occurrence of opportunistic infections and neoplasms. The neuropathological abnormalities resulting from either the direct effects of virus in the brain [1]–[3] or due to virotoxins (viral proteins/inflammatory mediators) released from infected cells in the central nervous system (CNS) [4], [5] are collectively termed as HIV-associated neurocognitive disorders (HAND). HAND comprises of a series of neurological impairments ranging from asymptomatic neurocognitive impairment (ANI), to minor neurocognitive disorder (MND), to the most severe manifestation of HAND, HIV-associated dementia (HAD) [6]. Though there is no evidence of infection of neurons by HIV, neuronal damage associated with HAD has most commonly been observed in the basal ganglia, hippocampus, deep white matter and frontal cortex leading to motor, cognitive and behavioral abnormalities [7].

Severe neuropathological changes leading to higher neurocognitive impairment have sometimes been linked to advanced immunosuppression either due to co-morbidity with other viral infections such as hepatitis C virus (HCV) [8], [9] or due to illicit drug abuse [10]–[12]. Immunosuppression by opiates, particularly morphine, has been very well studied and is mediated via alterations in peripheral lymphocyte activity [13] and suppression of major histocompatibility complex-II (MHC-II) expression [14]. Morphine renders the brain more susceptible to HIV infection by enhancing macrophage infiltration by inducing MCP-1 expression from activated astrocytes and resident microglia [15]. Morphine and cocaine mediated changes in expression of various tight junction proteins leads to altered blood-brain barrier permeability, thereby facilitating virus entry into the brain [16], [17]. Enhancement of simian/human immunodeficiency virus (SHIV) replication in cerebral compartments of chronic morphine exposed rhesus macaques [18] also provides evidence for greater neuropathological changes seen in HIV/AIDS patients abusing illicit substances. However, the molecular mechanisms underlying opioid mediated enhancement of HIV neuropathogenesis are still unknown and warrant extensive explorations.

The widespread use of highly active anti-retroviral therapy (HAART) has altered the spectrum of neurocognitive impairments categorized in HAND. Although the incidence of HAD, the most severe manifestation of HAND, has decreased significantly, the overall prevalence of HAND is still reported in 40-50% of HIV/AIDS patients [6], [19], [20]. The persistence of milder forms of HAND in HIV/AIDS patients undergoing HAART has been partly attributed to the longer life expectancy of the patients [21]. Problems arising due to resistance to HAART drugs and toxicities associated with their long-term exposure has also complicated the later stages of the lives of HIV/AIDS patients [22], [23]. CNS inflammation resulting from aberrant immune activation following HAART is also a matter of serious consideration [24]. These complications have led to extensive search for neuroprotectants with potential to be used as adjuncts to the current HAART. Falling in this category are drugs that can target the suspected pathways of neuronal injury, which include anti-inflammatory compounds, anti-oxidants, N-methyl-D-aspartate receptor (NMDAR) antagonists and calcium channel blockers [25], [26]. Neurotrophic factors such as brain derived neurotrophic factor (BDNF), nerve growth factor (NGF) and glial cell line-derived growth factor (GDNF) have already been helpful in protection against various neurotoxins [27]–[29] and are being speculated for their possible use as neuroprotective-adjuncts for HAART. Some studies have focused on platelet derived growth factor-BB (PDGF-BB) mediated neuroprotection against HIV proteins Tat [30] and gp120 [31] making it a prime candidate for further explorations. However for cases involving combined HIV/AIDS and drug exposure, that have a more devastating effect on the brain, detailed studies are lacking and therefore extensive investigations are warranted in this area.

We thus investigated the molecular mechanisms involved in morphine-induced exacerbation of HIV-Tat toxicity in human neurons and human neuroblastoma cells and further explored whether a neurotrophic factor, such as PDGF-BB could rescue neurons from the combined damage inflicted by these toxic agents. We demonstrated the involvement of mitogen-activated protein kinase (MAPK) extracellular signal-regulated kinase-1/2 (ERK1/2) and c-Jun N-terminal kinase (JNK) pathways in mediating the toxicity induced by Tat and morphine and also observed significant neuroprotective effects of PDGF-BB against HIV-Tat and morphine mediated combined toxicity. In addition to this, we found that this protection was mediated by phosphatidylinositol–3 kinase (PI3K)/Akt pathway.

## Results

### Morphine enhances HIV-Tat induced toxicity in primary human neurons and neuroblastoma cells

**Figure 1:**
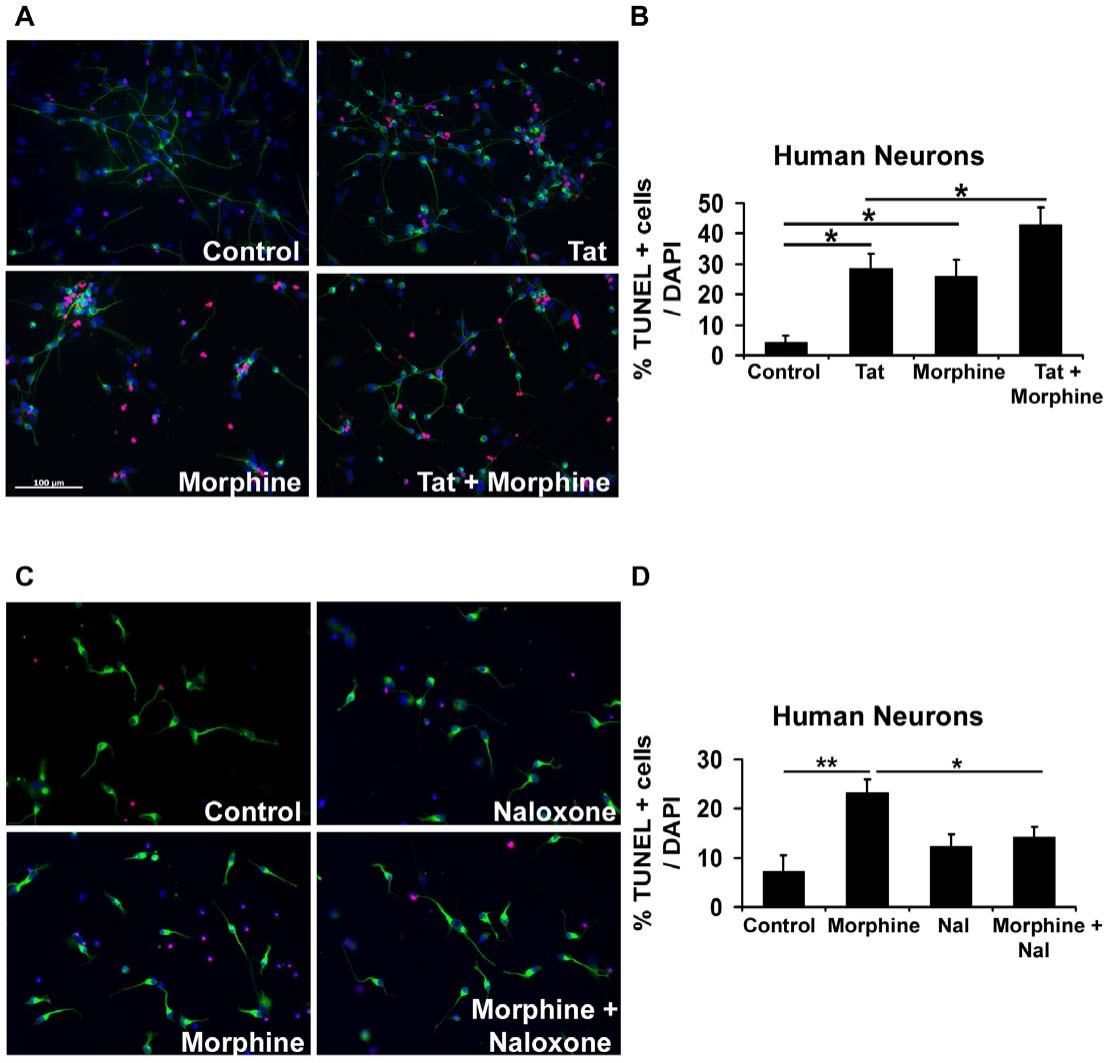
Morphine enhances HIV-Tat induced toxicity in human neurons: Involvement of opioid receptors. (A) Neurons differentiated from human neural precursor cells were treated with 100 ng/ml Tat and/or 100 nM morphine for 24 hours and apoptosis was detected by TUNEL assay. Neuronal cultures were also immunostained for β-III tubulin, a neuronal marker, and labeled with FITC conjugated secondary antibody (green). (B) Represents quantitative assessment of the apoptosis induced by Tat and/or morphine in neurons as shown in (A). Morphine significantly increased Tat induced damage to human neurons as depicted by a higher number of TUNEL positive (red/pink) apoptotic cells than when either of the agents was present independently. (C) To determine opioid receptor involvement in morphine-induced toxicity, human neurons were pre-treated with 1 µM naloxone, a general opioid receptor antagonist, prior to the addition of 100 nM morphine. After 24 hours, apoptosis was detected by TUNEL assay. (D) Represents quantitative assessment of human neurons treated with morphine in presence or absence of naloxone as shown in (C). Naloxone significantly inhibited morphine induced toxicity indicating opioid receptor involvement. Data represents mean ± standard deviation from 3 independent experiments. * − p<0.05. Scale bar denotes 100 µm.

**Figure 2:**
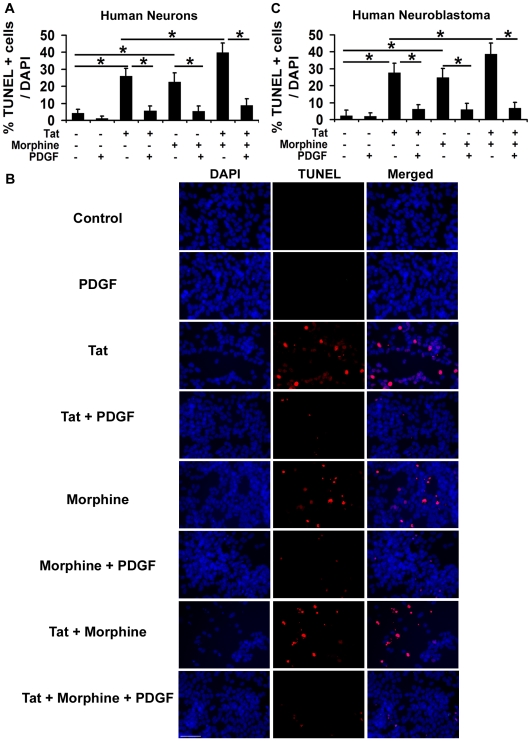
PDGF-BB protects neurons against Tat and morphine induced apoptosis. (A) Human neurons were exposed to 100 ng/ml Tat and/or 100 nM morphine with or without prior treatment with 40 ng/ml PDGF-BB. After 12 hours, apoptosis was detected by TUNEL assay. Quantitative assessment of TUNEL positive (red) apoptotic cells indicates that Tat and morphine together elicited far greater toxicity than either of these agents alone, as previously observed. With the addition of PDGF-BB, Tat and morphine induced toxicity was completely reversed as seen by lesser number of TUNEL positive cells. Also, PDGF-BB abrogated the toxicity subjected by either of the two agents alone. (B) Similar experiments were performed in human neuroblastoma cells and PDGF-BB was found to be protective against the toxicity elicited by Tat and morphine in these cells as well. (C) Represents quantitative assessment of the images shown in (B) for the PDGF-BB mediated protection against Tat and morphine in human neuroblastoma cells. Data represents mean ± standard deviation from 4 independent experiments. * − p<0.05, ** − p<0.005. Scale bar denotes 50 µm.

To assess the toxicity induced by Tat and morphine, human neurons were exposed to 100 ng/ml Tat and/or 100 nM morphine for 24 hours and apoptosis was detected by TUNEL assay (Figure 1A). Untreated neurons exhibited 4.24% apoptosis as indicated by red TUNEL positive cells that appear pink in the figure composite due to co-localization with DAPI stained blue nuclei of the cells. Whereas Tat exposure by itself resulted in 28.63% apoptotic neurons, in combination with morphine, apoptosis was significantly enhanced to 42.88% (Figure 1B). Neurons treated only with morphine exhibited 25.97% apoptosis. Similar results were obtained when human neuroblastoma cells were treated with Tat and/or morphine (Figure 2B and 2C). Morphine is known to act primarily via mu opioid receptors (MOR) [32], although its effect on delta and kappa opioid receptors cannot be ignored. We first examined the expression of mu opioid receptors on human primary neurons and neuroblastoma cells and observed that MOR are present on them. To confirm that morphine-induced toxicity was mediated via opioid receptors, we pre-treated human neurons with naloxone, which is a general opioid receptor antagonist. As shown in Figure 1C and 1D, pre-treatment with naloxone significantly reduced morphine-induced toxicity in human neurons, indicating that morphine's effect is mediated via opioid receptors. Similar effects of naloxone were also observed in human neuroblastoma cells (data not presented).

### PDGF-BB protects neurons against Tat and morphine induced apoptosis

PDGF-BB exerts neuroprotective effects against HIV proteins gp120 and Tat [30], [31]. However it is not known if PDGF-BB can protect neurons against the combined Tat and morphine induced toxicity as drug abuse is quite common in HIV/AIDS patients. To determine this, we pre-treated differentiated human neurons with 40 ng/ml PDGF-BB for 30 minutes prior to addition of Tat and morphine. As shown in Figure 2A, neurons treated with both Tat and morphine together resulted in 39.73% TUNEL positive cells and PDGF-BB pre-treatment offered a significant protection against these two agents, decreasing TUNEL positive apoptotic cells to 8.64%. We also observed a significant decrease in apoptosis induced by either of these agents independently with the addition of PDGF-BB (Figure 2A). Similar experiments were performed with human neuroblastoma cells and it was observed that PDGF-BB was also able to abrogate Tat and morphine induced damage to these cells (Figure 2B and 2C). To further explore if PDGF-BB mediated protection is specific to Tat and morphine induced toxicity, we exposed human neurons to etoposide (10 µM), which is known to induce apoptosis via DNA damage [33], with or without pre-treatment with PDGF-BB. After 24 hours of treatment, etoposide treated cells showed more than 4 fold increase in apoptosis over control (untreated) cells, as was determined by TUNEL assay (Etoposide treated neurons showed 43.69% TUNEL positive cells over DAPI, whereas the same was 9.48% in control neurons). PDGF-BB pre-treatment given to neurons before exposing them to etoposide did not block the apoptosis induced by the agent (39.28% TUNEL positive cells over DAPI), hence confirming the specificity of PDGF-BB against Tat and morphine toxicity.

### PDGF-BB maintains the fine balance between pro-apoptotic and anti-apoptotic proteins in human neurons exposed to Tat and morphine

**Figure 3:**
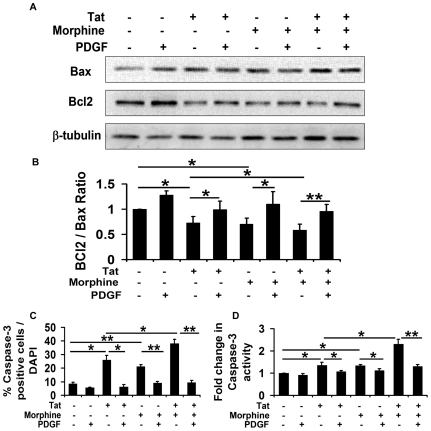
PDGF-BB maintains Bcl2/Bax ratio and decreases caspase-3 activation induced by Tat and morphine. (A) Human neurons were treated with 100 ng/ml Tat and/or 100 nM morphine for 12 hours with or without pre-treatment with 40 ng/ml PDGF-BB. Proteins were isolated and levels of key anti-apoptotic and pro-apoptotic proteins, Bcl2 and Bax, respectively were determined by Western blotting. (B) Densitometric analysis of the blots shown in (A). Tat and morphine together resulted in a significantly decreased ratio of Bcl2/Bax, indicating cell's progress towards apoptosis. PDGF-BB pre-treatment, however, resulted in maintenance of the Bcl2/Bax ratio, inhibiting apoptotic changes in the cells and rescuing them from Tat and morphine induced toxicity. (C) Human neuroblastoma cells were treated with 100 ng/ml Tat and/or 100 nM morphine for 12 hours and caspase-3 activity was studied using Immunocytochemistry for cleaved caspase-3. Quantitative assessment of the images captured from at least five random fields/experimental group demonstrates that Tat and morphine together resulted in a significantly more number of cells positive for cleaved caspase-3 (green) than independently with either Tat or morphine. Also, PDGF-BB pre-treatment was able to rescue cells from the toxicity induced by either Tat or morphine alone, or in combination, as fewer number of cells positive for cleaved caspase-3 were observed. (D) Caspase activation was also studied using a colorimetric assay for caspase-3 activity. Quantitative assessment reveals more than 2 fold increase in caspase-3 activity in cells treated with both Tat and morphine, which was significantly higher than either of the agents alone. PDGF-BB pre-treatment resulted in abrogation of the Tat and morphine induced activation of caspase-3, thereby protecting the cells from apoptotic death. Data represents mean ± standard deviation from 3 independent experiments. * − p<0.05; ** − p<0.005.

Bcl2 family members are the major regulators of mitochondrial integrity and control mitochondria-initiated cytochrome c and caspase activation [34], [35]. The Bcl2 family includes anti-apoptotic proteins such as Bcl2 and Bcl-XL and pro-apoptotic proteins such as Bax, Bak, Bim etc. Thus levels of these anti- and pro-apoptotic proteins were analyzed in human neurons exposed to Tat and morphine. As shown in Figure 3A and 3B, after 12 hours of treatment, there was a significant decrease in the expression of Bcl2 in neurons treated with both Tat and morphine as opposed to cells treated with only one toxic agent, which decreased the Bcl2/Bax ratio indicating that cells were progressing towards apoptosis (Figure 3B). PDGF-BB pre-treatment prevented this tilting of cell's balance towards apoptosis by promoting Bcl2 and inhibiting Bax expression. Beta tubulin expression was probed to ensure equal loading of samples and served as an input control.

### PDGF-BB prevents caspase-3 activation induced by Tat and morphine in neuroblastoma cells

To substantiate the findings that Tat and morphine induced toxicity to neuroblastoma cells involved apoptotic pathways, we next studied caspase-3, a protease that is essential for neuronal apoptosis [36]. Human neuroblastoma cells were treated with 100 ng/ml Tat and/or 100 nM morphine with or without pre-treatment with 40 ng/ml PDGF-BB. As shown in Figure 3C, immunocytochemical analysis for caspase-3 indicated that 12 hours post-treatment Tat and morphine together resulted in 35.88% of cells positive for cleaved caspase-3, whereas Tat and morphine alone exhibited only 22.97% and 19.63% cleaved caspase-3 positive cells respectively. PDGF-BB pre-treatment significantly attenuated caspase-3 activation, reducing cleaved caspase-3 positive cells to 7.98% in Tat alone, 7.53% in morphine alone and 8.98% when both these agents were present together (Figure 3C).

Caspase-3 involvement was further confirmed by a colorimetric caspase-3 activity assay (Figure 3D) which showed a substantial 2.3 fold increase in caspase-3 activity by Tat and morphine together, whereas Tat and morphine alone exhibited only 1.36 and 1.33 fold increase in caspase-3 activity respectively. As expected, PDGF-BB abrogated caspase-3 activation in all the treatment groups. Thus, PDGF-BB protects human neuroblastoma cells by preventing Tat and morphine induced caspase-3 activation.

### PDGF-BB averts Tat and morphine induced oxidative stress in human neuroblastoma cells

**Figure 4:**
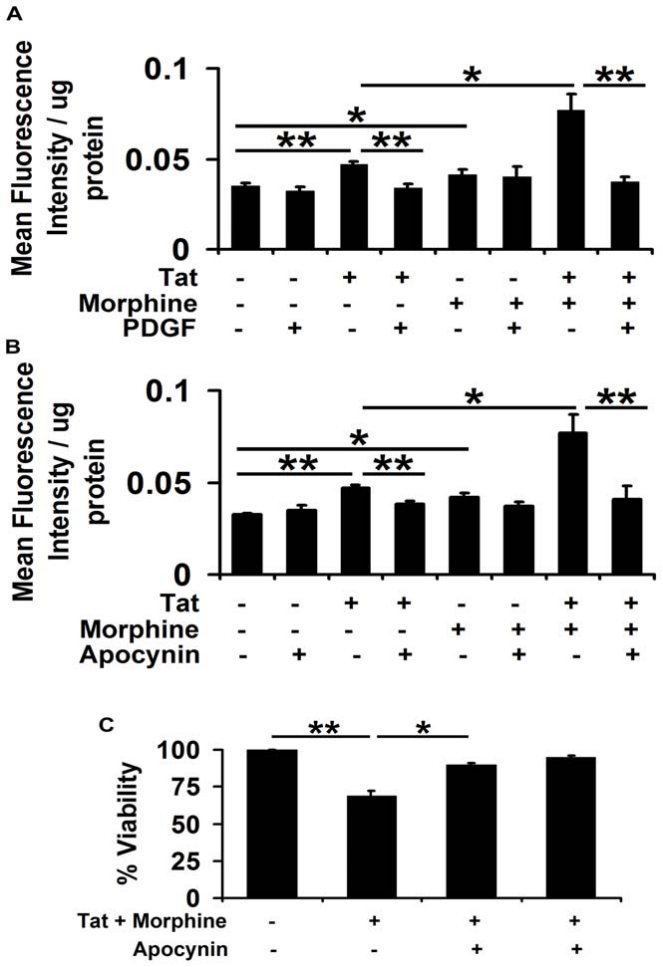
PDGF-BB averts Tat and morphine induced reactive oxygen species generation in human neuroblastoma cells. (A) Human neuroblastoma cells were exposed to 100 ng/ml Tat and/or 100 nM morphine with or without pre-treatment with 40 ng/ml PDGF-BB. After 3 hours, oxidative stress in the cells was determined by DCFDA oxidation assay. Quantitative assessment indicates significant elevation in oxidative stress levels in cells treated with Tat and morphine as compared to cells treated with either of the agents alone. No such oxidative stress was observed in the cells pre-treated with PDGF-BB prior to Tat and morphine exposure. (B) Inhibition of NADPH oxidase by pre-treatment of cells with 250 µM apocynin before exposing to Tat and morphine resulted in significant abrogation of reactive oxygen species generation, indicating the involvement of NADPH oxidase in ROS generation. (C) Tat and morphine induced toxicity in human neuroblastoma cells was mediated by oxidative stress as inhibition of ROS generation by NADPH oxidase inhibitor, apocynin, resulted in significant abrogation of this toxicity. Data represents mean ± standard deviation from 4 independent experiments. * − p<0.05; ** − p<0.005.

Reactive oxygen species (ROS) have been suggested as possible mediators of cell damage in response to HIV-Tat [37]. Chronic morphine is also a well-known inducer of ROS in SHSY5Y cells [38]. To determine the levels of oxidative stress induced by the combined exposure of both these toxic agents, human neuroblastoma cells were subjected to Tat and/or morphine for 3 hours with or without pre-treatment with PDGF-BB. As shown in Figure 4A, Tat and morphine in combination showed more than 2 fold increase in ROS production, which was significantly higher than both these agents alone. Interestingly, pre-treatment with PDGF-BB prevented ROS generation due to Tat alone and also when Tat is in combination with morphine (Figure 4A).

NADPH oxidase is a membrane protein that has been shown to regulate oxidative changes in the cell in response to various extra cellular stimuli [39], [40]. To determine whether ROS generation by Tat and morphine involved NADPH oxidase, we pre-treated human neuroblastoma cells with 250 µM apocynin for 1 hour prior to exposing them to Tat and morphine. As shown in Figure 4B, inhibition of NADPH oxidase by apocynin resulted in significant abrogation of ROS induced by Tat and morphine. Since reactive oxygen species have been shown to regulate early steps of neuronal apoptosis [41], we next sought to determine if alleviating ROS production could rescue the cells from Tat and morphine induced toxicity. Interestingly, we observed that one-hour pre-treatment with apocynin followed by exposure to Tat and morphine significantly inhibited the toxicity induced by these agents, suggesting NADPH mediated ROS production as a possible mediator of Tat and morphine induced toxicity (Figure 4C).

### PDGF-BB prevents mitochondrial membrane depolarization in human neurons exposed to Tat and morphine

**Figure 5:**
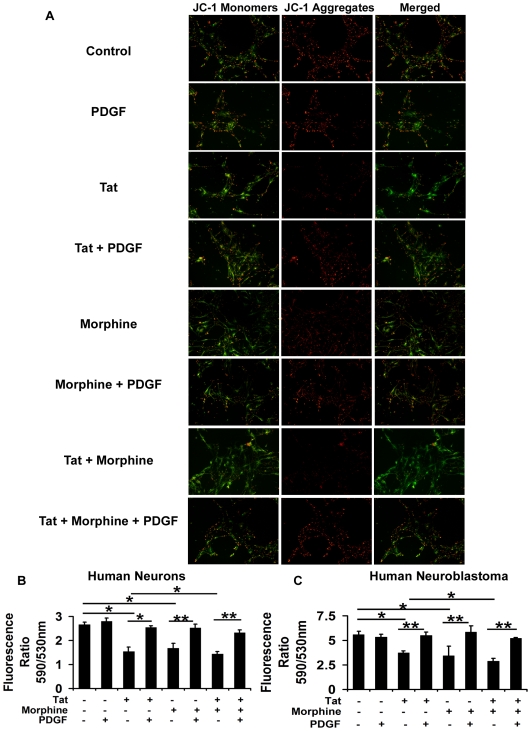
PDGF-BB prevents Tat and morphine induced mitochondrial membrane depolarization in human neurons and neuroblastoma cells. (A) Human neurons were treated with 100 ng/ml Tat and/or 100 nM morphine with or without pre-treatment with 40 ng/ml PDGF-BB. After 12 hours, mitochondrial membrane depolarization was studied by JC-1 dye assay. Significant depolarization of the mitochondrial membrane was observed in neurons treated with both Tat and morphine as seen by presence of green JC-1 monomers. PDGF-BB maintained the mitochondrial integrity as indicated by the presence of red JC-1 aggregates in PGDF-BB pre-treated Tat and morphine exposed cells (B) From parallel experiments, quantitative assessment of mitochondrial membrane depolarization in human neurons was done and the ratio of JC-1 aggregates/monomers was calculated and represented. (C) Represents quantitative assessment of mitochondrial membrane depolarization in human neuroblastoma cells treated with Tat and morphine with or without PDGF-BB pre-treatment. Data represents mean ± standard deviation from 3 independent experiments. * − p<0.05; ** − p<0.005.

Apoptotic stimuli are known to cause changes in the inner mitochondrial membrane leading to opening of the mitochondrial permeability transition (MPT) pores and loss of the mitochondrial trans-membrane potential [42]–[44]. Tat and other viral proteins are also known to cause mitochondrial membrane depolarization [45]–[47]. To determine if morphine mediated aggravation of Tat toxicity also involved mitochondrial membrane disruptions, we treated human neurons with both these agents in combination and independently with or without pre-treatment with PDGF-BB (Figure 5A). As shown in Figure 5B, quantitative assessment of the mitochondrial membrane depolarization after 12 hours of treatment indicated a significant depolarization of the neuronal mitochondrial membrane by Tat and morphine as indicated by presence of green JC-1 monomers and this depolarization was significantly greater than what was induced by either of these agents independently. Mitochondrial membrane integrity was maintained in neurons treated with PDGF-BB prior to exposure of Tat and morphine as indicated by the presence of red JC-1 aggregates (Figure 5A and 5B). PDGF-BB was also able to conserve mitochondrial integrity with either of the toxic agents alone. Similar results were obtained when human neuroblastoma cells were treated with Tat and/or morphine with or without pre-treatment with PDGF-BB (Figure 5C).

### Involvement of MAPK pathways in Tat and morphine induced toxicity in human neuroblastoma cells

**Figure 6:**
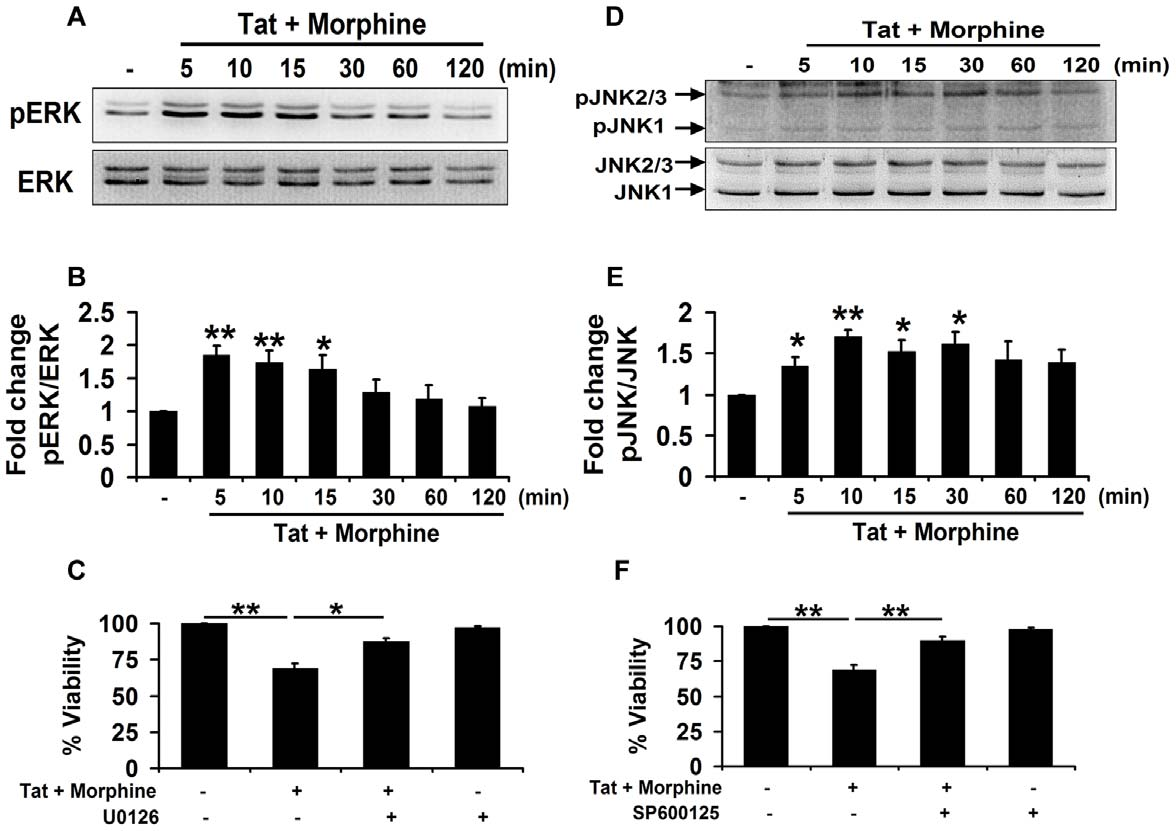
Involvement of c-Jun N-terminal kinase in Tat & morphine induced toxicity in human neuroblastoma cells. Human neuroblastoma cells were exposed to 100 ng/ml Tat and 100 nM morphine for the indicated times and activation of MAPK pathways was studied by Western blotting. (A) Tat and morphine induced a rapid and time-dependent increase in phosphorylation of ERK1/2 as shown by representative blots. (B) Densitometric analysis of the blots represented in (A) for activation of ERK1/2 as assessed by pERK/ERK. (C) Cell viability analysis (MTT) of human neuroblastoma cells pre-treated with MEK1/2 inhibitor, U0126, for 1 hour followed by exposure to Tat and morphine for another 12 hours led to significant abrogation of toxicity induced by these agents. (D) Phosphorylation of JNK following exposure to 100 ng/ml Tat and 100 nM morphine was studied by Western blotting for the times indicated as shown by the representative blots. (E) Densitometric analysis of the blots represented in (D) for phosphorylation of JNK as assessed by pJNK/JNK. (F) Inhibition of JNK activity by specific inhibitor, SP600125, led to the inhibition of Tat and morphine induced toxicity as shown by MTT assay. Data represents mean ± standard deviation from 3 independent experiments. * − p<0.05; ** − p<0.005.

To determine the molecular pathways involved in Tat and morphine induced toxicity in human neuroblastoma cells, a detailed investigation of MAPK pathways was done. Exposure of Tat and morphine resulted in a rapid and time-dependent increase in phosphorylation of extracellular signal-regulated kinase-1/2 (ERK1/2) as assessed by pERK/ERK (Figure 6A and 6B). To further probe into the individual roles of Tat and morphine in inducing ERK1/2 activation, SHSY5Y cells were treated independently with the two agents for the indicated times (Figure S1). Whereas Tat alone caused a robust activation of ERK1/2, exposure to morphine resulted in a lesser activation, which was observed only at 5 min post-treatment (Figure S1A). The ERK1/2 activation with morphine was blocked by using opioid receptor antagonist, naloxone, confirming their involvement (Figure S1B). The role of ERK1/2 in mediating Tat and morphine induced toxicity was further confirmed by using MEK1/2 inhibitor, U0126. Inhibition of ERK1/2 phosphorylation by specific MEK1/2 inhibitor, U0126 partially rescued the cells from the damage inflicted by Tat and morphine as assessed by MTT assay (Figure 6C).

Tat and morphine exposure also resulted in a time dependent activation of c-Jun N-terminal Kinase (JNK) as assessed by pJNK/JNK levels (Figure 6D and 6E). As shown in Figure S1C, Tat and morphine independently resulted in distinct activation patterns for JNK. Whereas Tat caused rapid phosphorylation of JNK, morphine caused a more delayed but sustained JNK activation (Figure S1C). Furthermore we found that the JNK activation in the Tat and morphine group was significantly higher in comparison to the untreated control at all time points. The Tat and morphine group was also significantly higher in comparison to the Tat alone group at 30 min and the morphine alone group at 10 min post-treatment (Figure S1C). Morphine induced JNK activation was also found to be opioid receptor mediated as opioid receptor antagonist, naloxone, significantly blocked the morphine induced JNK activation (Figure S1D). Inhibition of JNK signaling by specific JNK inhibitor SP600125 prior to exposing SHSY5Y cells to Tat and morphine, resulted in amelioration of Tat and morphine induced toxicity as assessed by MTT assay (Figure 6F).

However, investigation of the p38-MAPK pathway did not show any increase in phosphorylation of p38 in our system (data not shown). To confirm this, Tat and morphine induced toxicity to human neuroblastoma cells was analyzed after inhibiting the p38 pathway by using a specific p38 inhibitor, SB203580. Inhibition of the p38 pathway did not lead to any significant abrogation of Tat and morphine induced toxicity, thus negating its involvement in the process (data not shown). Therefore, ERK1/2 and JNK, but not p38 were found to be involved in mediating Tat and morphine induced toxicity in human neuroblastoma cells.

### PDGF-BB mediated protection against Tat and morphine involves PI3K pathway

**Figure 7:**
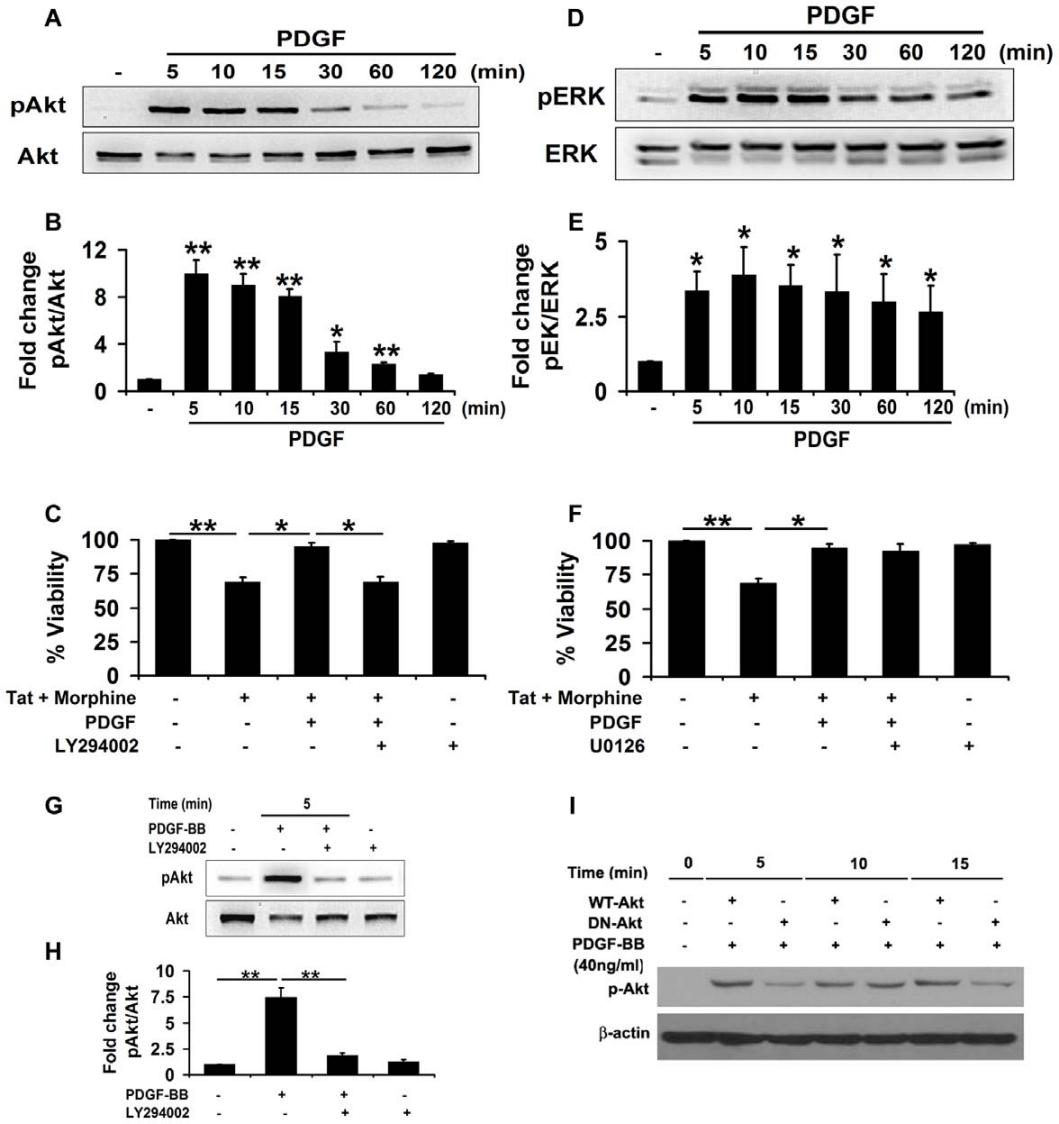
Involvement of PI3K/Akt in PDGF-BB mediated protection against Tat and morphine. (A) Human neuroblastoma cells were exposed to 40 ng/ml PDGF-BB for the indicated times and activation of Akt was studied by Western blotting. (B) Densitometric analysis of the blots represented in (A) for activation of Akt, as indicated by pAkt/Akt. (C) Cell viability analysis (MTT) of human neuroblastoma cells pre-treated with PI3K inhibitor, LY294002, for 1 hour followed by exposure to PDGF-BB and Tat-morphine led to significant abrogation of PDGF-BB mediated protection against Tat and morphine. (D) ERK1/2 phosphorylation following exposure to 40 ng/ml PDGF-BB was studied by Western blotting for the time-points indicated and an increase in ERK1/2 activation was noted. (E) Densitometric analysis of the blots represented in (D) for phosphorylation of ERK1/2 as indicated by pERK/ERK. (F) Inhibition of MEK1/2 activity by specific inhibitor, U0126, did not lead to reversal of PDGF-BB mediated protection against Tat and morphine as shown by MTT assay. (G) Inhibition of the PI3K pathway by LY294002 led to abrogation of PDGF-BB mediated activation of Akt in neuroblastoma cells, confirming its role in PDGF-BB mediated protection. (H) Densitometric analysis of the blots represented in (G) for Akt activation as represented by pAkt/Akt. (I) Role of Akt was further confirmed when SHSY5Y cells infected with DN-Akt showed reduced activation of Akt with PDGF-BB exposure. For normalization, the same blot was re-probed for beta-actin. Data represents mean ± standard deviation from 3 independent experiments. * − p<0.05; ** − p<0.005.

PDGF-BB has been shown to regulate cell survival, proliferation and differentiation via its diverse effects on both PI3K/Akt and MAPK/ERK pathways [48], [49]. Some recent studies have also implicated an important role of PI3K/Akt in PDGF-BB-mediated protection against HIV protein, gp120 [50]. We thus investigated the role of Akt, which is downstream of PI3K in PDGF-BB induced signaling in human neuroblastoma cells. As shown in Figure 7A and 7B, PDGF-BB treatment caused a robust activation (more than 8 fold) of Akt as determined by pAkt/Akt levels in human neuroblastoma cells and this effect lasted for at least 60 minutes. To assess the role of PI3K in PDGF-BB mediated protection against Tat and morphine, we also used a specific PI3K inhibitor, LY294002. As shown in Figure 7C, inhibition of PI3K signaling by LY294002 led to loss of PDGF-BB mediated abrogation of toxicity induced by Tat and morphine, confirming that PI3K is a key player in PDGF-BB mediated protection against Tat and morphine. The role of Akt in PDGF-BB mediated neuroprotection was further strengthened as LY294002 mediated inhibition of PI3K pathway led to amelioration of PDGF-BB induced Akt activation in neuroblastoma cells (Figure 7G and 7H). To confirm this, SHSY5Y cells were infected with adenoviral constructs containing wild-type (WT-Akt) or dominant-negative Akt (DN-Akt) and subsequently treated with PDGF-BB. As shown in Figure 7I, neuroblastoma cells containing DN-Akt showed substantial reduction in PDGF-BB induced Akt activation, hence confirming the role of Akt in PDGF-BB mediated enhancement of cell survival.

Analysis of the MAPK/ERK pathway revealed that PDGF-BB exposure also led to a sustained (>2 fold) increase in ERK1/2 activity as determined by pERK/ERK (Figure 7D and 7E). However, pre-treatment of cells with a specific MEK1/2 inhibitor, U0126, followed by exposure to PDGF-BB and Tat-morphine did not lead to inhibition of PDGF-BB mediated protection against these two agents (Figure 7F), which suggests that PDGF-BB mediated abrogation of Tat and morphine induced toxicity was perhaps not mediated via the MAPK/ERK pathway.

## Discussion

In spite of ample evidence suggesting increased rate of progression and severity of HAD with opioid abuse, the exact stages of interaction between HIV and drugs of abuse are still not well understood. One of the major obstacles in delineating the cellular and molecular pathways for the enhanced neurodegeneration in drug abusing HIV/AIDS patients is the unavailability of good animal models for studying HIV neuropathogenesis [51]. Few *in vivo* models have been tried but with limited success [52]–[55]. Though *in vivo* studies accurately mimic the biological response to a pathogen, *in vitro* studies have their own advantage, being an extensively explorative tool to delineate the molecular mechanisms behind every aspect of cellular response to a stimulus and are extensively used for neuroAIDS studies [56].

To study the co-morbid effects of Tat and morphine, we used an *in vitro* model system of human neurons differentiated from human neural precursor cells isolated from fetal brains. In addition to human neurons, we relied on human neuroblastoma cells, SHSY5Y, for studies related to elucidation of molecular pathways, as obtaining human neurons in large numbers is quite difficult. We used 100 ng/ml Tat and 100 nM morphine for our studies and obtained identical results in both the cell types. The doses of Tat, morphine and PDGF-BB used in the study were in concordance with the current literature on the subject [31], [57], [58]. Our study clearly demonstrated the role of morphine in enhancing Tat induced toxicity in human neurons as well as in human neuroblastoma cells, signifying their co-morbid effects and possibly intercommunication at multiple steps. We thoroughly examined the underlying mechanisms at cellular and molecular levels and obtained several interesting results that we have presented in this paper.

In accordance with the previous literature demonstrating the enhanced toxicity of HIV proteins in the presence of opioids, particularly morphine [59], [60], we also observed a significant increase in Tat toxicity in the presence of morphine, in human neurons. Following 24 hours of treatment, Tat and morphine independently induced apoptosis in human neurons as assessed by TUNEL assay, the toxicity however, was further increased when both these agents were present together as observed by increased number of TUNEL positive apoptotic neurons. Similar results depicting morphine-induced exacerbation of Tat toxicity were also obtained when human neuroblastoma cells were exposed to Tat and/or morphine for 12 hours. We used different time-points for assessment of apoptosis in the two cell types since 24-hour treatment of SHSY5Y cells led to detachment of cells from the surface. We observed approximately 3 to 5 fold increase in toxicity in these cells with morphine exposure, which was surprising, but the same was consistent across a number of experiments repeated throughout the study. Some groups have reported such toxicities but with chronic treatments [61]–[63].

Although the effects of morphine are generally thought to be mediated via mu opioid receptors, involvement of delta and kappa opioid receptors at higher concentrations of morphine cannot be ruled out. We therefore examined and confirmed by immunocytochemistry that these cells expressed the opioid receptors. Furthermore, we also observed that morphine-mediated apoptosis in human neurons and SHSY5Y cells was mediated through opioid receptors since pre-treatment of these cells with naloxone, a general opioid receptor antagonist, abrogated the morphine-induced toxicity.

Several studies have reported neurotrophic factor mediated protection against HIV proteins Tat and gp120 [27], [28], however there are no available reports where protection against co-morbidity, particularly drug enhanced toxicity of HIV proteins has been analyzed. PDGF-BB has been shown to protect SHSY5Y cells against Tat by modulating extracellular glutamate and intracellular calcium levels [30]. Among its different isoforms, PDGF-BB has been shown to be the most effective in promoting neuronal survival [64], [65]. We next wanted to explore whether PDGF-BB could also rescue human neurons and SHSY5Y cells from the enhanced toxicity induced by Tat and morphine together. PDGF-BB pre-treatment resulted in a significant reduction in apoptotic death induced by simultaneous exposure of Tat and morphine when assessed by TUNEL assay. We also found complete abrogation of the toxicity induced by either of these agents independently with the addition of PDGF-BB. Our study thus provides convincing evidence that PDGF-BB extends its protective effect against both Tat and morphine induced toxicity. Interestingly, PDGF-BB was not able to protect against the apoptotic death caused by etoposide exposure in neurons, implying the specificity of PDGF-BB against Tat and morphine induced toxicity.

Apoptotic events in the cells are governed by levels of anti-apoptotic (Bcl-XL family) and pro-apoptotic proteins (Bax, Bak). Decreased Bcl2/Bax ratio leads to the formation of mitochondrial membrane permeability transition pores and subsequent release of various apoptosis mediators such as cytochrome c, an activator of the caspase cascade [34], [35]. To corroborate the findings of apoptotic rescue by PDGF-BB against Tat and morphine toxicity, we next analyzed the relative levels of anti- and pro-apoptotic proteins, Bcl2 and Bax, respectively. Significant reduction in Bcl2/Bax ratio was observed in human neurons treated with Tat and morphine, indicating the involvement of apoptotic pathway. The decrease in Bcl2/Bax ratio in neurons exposed to both Tat and morphine was greater than what was induced by either agent alone. Furthermore, the alterations in the Bcl2/Bax ratio were abrogated with pre-treatment of cells with PDGF-BB, indicating the maintenance of levels of anti- and pro-apoptotic proteins in the cells favoring neuroprotection. Subsequent analysis of mitochondrial membrane integrity by fluorescent JC-1 dye revealed extensive depolarization of the mitochondrial membrane in human neurons exposed to Tat and morphine. PDGF-BB pre-treatment of neurons resulted in the maintenance of mitochondrial membrane potential even in the presence of Tat and morphine as indicated by JC-1 aggregates that appeared red. Thus PDGF-BB was able to preserve mitochondrial membrane integrity in human neurons subjected to Tat and morphine exposure. Similar results were obtained when SHSY5Y cells were treated with Tat and morphine. Finally involvement of caspase-3 in Tat and morphine induced apoptosis was studied in SHSY5Y cells since caspases have been recognized as prime mediators of neuronal apoptosis in developmental and neuropathological conditions [66]. Using a colorimetric caspase-3 activity assay and immunostaining for cleaved caspase-3, we observed a concomitant increase in caspase-3 activity in cells exposed to both the toxic agents, which was significantly higher than the caspase-3 activation with either agent alone, substantiating our previous data of enhanced apoptosis in the cells subjected to co-exposure of Tat and morphine. To confirm that the effect was caspase-3 specific, we also included an irreversible caspase-3 inhibitor, Z-VAD-FMK, in our study along with Tat and morphine. Interestingly, PDGF-BB pre-treatment was able to prevent caspase-3 activation in response to Tat and morphine and hence rescued the cells from apoptosis. This is in agreement with a previous study where PDGF-BB has been shown to inhibit caspase-3 activity in SHSY5Y cells [31].

Several reports have shown that Tat induced cell damage involves reactive oxygen species (ROS) generation [37]. Excessive production of ROS leads to a state of oxidative stress in the cells that can also initiate programmed cell death [67]. Combined exposure of Tat and morphine in SHSY5Y cells led to a dramatic increase in ROS generation, which was significantly higher than the ROS levels generated by either agent alone. ROS generation was found to be NADPH dependent in SHSY5Y cells since apocynin, an inhibitor of NADPH oxidase, blocked Tat and morphine induced elevations in ROS levels. This was further confirmed as inhibition of NADPH oxidase led to abrogation of Tat and morphine induced toxicity and improved the viability of SHSY5Y cells. Also, pre-treatment with PDGF-BB resulted in complete abrogation of ROS production by Tat and morphine. These findings are in agreement with previous reports showing NADPH mediated ROS production in SHSY5Y cells in response to HIV proteins [68]. Moreover, certain host genetic factors like apolipoprotein E4 have also been shown to influence oxidative stress and neurotoxicity levels in response to Tat and opioid co-exposure [69]. Thus further explorations for factors that could affect HIV/HIV protein's pathology should be extensively undertaken.

MAPK signaling pathways have been shown to exert diverse effects on cell survival and death [70]. Activation of JNK, p38 and ERK MAPK pathways have been documented in mediation of various cell death signals from a wide variety of stimuli [38], [68], [71], [72]. Chronic high-dose morphine has been shown to decrease SHSY5Y cell viability via activation of JNK signaling [38]. Our experiments in the current study on MAPK pathways revealed the involvement of JNK and ERK1/2 pathway in Tat and morphine induced toxicity in SHSY5Y cells, since inhibition of these pathways by specific inhibitors led to abrogation of Tat and morphine mediated toxicity. Individual analysis of the effects of Tat and morphine on MAPK pathways revealed that there were not much significant changes in ERK1/2 activation when neuroblastoma cells were exposed to Tat in presence or absence of morphine, although co-exposure of morphine along-with Tat significantly enhanced duration of JNK activation. Also, morphine's effect was mostly mediated via the JNK pathway rather than the ERK1/2 pathway. Furthermore, morphine-induced activations in JNK and ERK1/2 pathways were completely blocked by naloxone, thereby indicating opioid receptor involvement. Interestingly when it comes to p38-MAPK signaling, HIV-Tat and morphine have been shown to exert opposing effects. For example, in U87 astrocytes Tat has been shown to activate p38 signaling [73], where-as SHSY5Y cells exposed to morphine do not show any p38 activation [38]. In our study, we also did not find any p38 activation in SHSY5Y cells following Tat and morphine exposure. This was also confirmed when a specific inhibitor of p38, SB203580, failed to rescue SHSY5Y cells from damage inflicted by Tat and morphine together.

Our experimental data also suggests that PDGF-BB mediated protection against Tat and morphine was mediated by the PI3K pathway since PDGF-BB caused significant activation of pro-survival signal Akt, the prime mediator of the PI3K pathway. This was further substantiated as specific PI3K inhibitor, LY294002, abrogated PDGF-BB mediated Akt activation and also led to loss of protection against Tat and morphine. Role of Akt was further substantiated when SHSY5Y cells infected with DN-Akt showed a marked reduction in PDGF-BB induced Akt activation. This is in agreement with a recent study which documented the involvement of PI3K/Akt in PDGF-BB mediated signaling [50]. Finally, we observed that ERK1/2 activation occurred after SHSY5Y cells were exposed to PDGF-BB, which was puzzling as inhibition of the ERK1/2 phosphorylation by specific MEK1/2 inhibitor did not alter PDGF-BB mediated protection against Tat and morphine, thus neglecting its role in PDGF-BB signaling. Similar observation of ERK1/2 activation in SHSY5Y cells upon PDGF-BB exposure but its lack of involvement in PDGF-BB mediated neuroprotection has been reported in a recent study [31]. Hence it was concluded that PDGF-BB mediated protection of SHSY5Y against Tat and morphine involved the PI3K pathway but not the ERK1/2 pathway.

In conclusion we believe this study provides new insights into the mechanism of morphine induced enhancement of Tat toxicity in human neurons and neuroblastoma cells and highlights the role of PDGF-BB as a neuroprotective agent, which could be considered for therapeutic interventions in HIV/AIDS-drug abuse cases. Further investigations on similar lines with *in vivo* models as well as post mortem human brain samples from drug abusing HIV/AIDS population are warranted.

## Materials and Methods

### Materials

Human neuroblastoma cells (SHSY5Y) were a kind gift from Dr. Anirban Basu (NBRC). Human recombinant PDGF-BB, PDGF-AB, BDNF, basic fibroblast growth factor (bFGF), epidermal growth factor (EGF), poly-D-lysine (PDL), Dulbecco's modified Eagle's medium (DMEM), morphine sulphate, specific PI3K inhibitor LY294002, etoposide and naloxone hydrochloride were all purchased from Sigma Aldrich (St. Louis, MO, USA). Neural survival factor-1 (NSF) was purchased from Cambrex (Charles City, IA, USA). Neurobasal and N2 supplement were purchased from Invitrogen (San Diego, CA, USA). The specific MAPK/ERK kinase-1/2 (MEK1/2) inhibitor U0126, p38 inhibitor SB203580, JNK inhibitor SP600125 and NADPH oxidase inhibitor apocynin were obtained from Calbiochem (San Diego, CA, USA). All other cell-culture reagents were purchased from Sigma Aldrich (St. Louis, MO, USA). Antibodies for pERK, ERK, pAkt, Akt, pJNK, JNK, Bax, Bcl2 and cleaved caspase-3 were all purchased from Cell Signaling (Danvers, MA, USA). Horseradish peroxidase conjugated secondary antibody and fluorescein isothiocyanate (FITC)-conjugated secondary antibody were purchased from Vector Laboratories (Burlingame, CA, USA).

### Methods

#### Ethics Statement

The National Brain Research Centre (NBRC) human ethics committee approved the project involving use of Human Fetal Brain Derived Neural Stem Cells as a Model for Studying Neurodegenerative Diseases. Fetal brain tissue was obtained from the aborted fetus after obtaining mother's written consent and the samples were processed as per the protocols approved by the NBRC Human Ethics Committee in compliance with the recommendations of the Indian Council of Medical Research, India.

#### Cell cultures and treatments

Human neurons were differentiated from primary human neural precursor cells isolated from 8- to 12- week-old fetus obtained from elective medical termination of first-trimester pregnancies performed at the local hospitals as described previously [58]. The handling of the aborted fetus for brain tissue was done after obtaining mother's written consent following protocols approved by the National Brain Research Centre (NBRC) Human Ethics Committee. Briefly, human neural precursor cells were isolated from the sub-ventricular zone of telencephalic region of the aborted fetuses and cultured in PDL coated flasks (Nunc, Kamstrupvej, Denmark) in neurobasal media supplemented with EGF, bFGF, NSF and N2 supplement. Neural precursor cells were then differentiated into neuronal lineage using neuronal media where the mitogenic factors, bFGF and EGF were replaced with BDNF and PDGF-AB, the rest of the contents remained same, as described earlier [74]. Media was half-replaced every alternate day and after three weeks cell purity was determined by immunocytochemistry using anti-beta-3 tubulin antibody (Covance, Berkeley, CA, USA) and were >98% pure. Human neurons were pre-treated with PDGF-BB (40 ng/ml) for 30 min followed by exposure to HIV-1B Tat (100 ng/ml) and/or morphine sulphate (100 nM) for 24 hours for assessment of apoptosis. Analysis of anti- and pro-apoptotic proteins (Bcl2 and Bax) and mitochondrial membrane integrity was done after exposing human neurons to Tat and morphine for 12 hours with or without pre-treatment with PDGF-BB for 30 minutes. All experiments with human neurons were done in complete neuronal media containing trophic factors (PDGF-AB and BDNF), as their withdrawal affected the viability of untreated human neurons.

Human neuroblastoma cells, SHSY5Y were cultured in DMEM supplemented with heat-inactivated fetal bovine serum (10% v/v) and 2 mM glutamine at 37°C in 5% CO_2_. SHSY5Y cells were serum-starved for 2 hours prior to treatment with PDGF-BB (40 ng/ml) for 30 min followed by exposure to HIV-1B Tat (100 ng/ml) and/or morphine sulphate (100 nM). Assessment of apoptotic death by TUNEL and measurement of cell viability by MTT assay was done after 12 hours of exposure to Tat and morphine with or without pre-treatment with PDGF-BB for 30 minutes. In the cell viability assays involving pharmacological inhibitors, cells were pre-cultured with the corresponding inhibitor for 1 hour prior to the treatment with PDGF-BB and/or HIV-Tat and morphine sulphate. In the WWestern blotting experiments involving pharmacological inhibitors, SHSY5Y cells were pre-treated either with LY294002 for 2 hours before addition of PDGF-BB (Figure 7G,H) or with naloxone for 1 hour before addition of morphine for the given treatment duration (Figure S1). Reactive oxygen species measurements were done after treating SHSY5Y cells with Tat and morphine for 3 hours with or without pre-treatment with PDGF-BB for 30 minutes.

#### Expression and purification of HIV-1 Tat

A mammalian expression vector of subtype B-Tat was prepared from the HIV-1 molecular clone YU-2, derived from brain of a HIV-1 AIDS dementia patient (GenBank Accession number M93258). The full-length (1–101 amino acid) recombinant Tat B protein was expressed in mammalian expression vector pET with His-Tag, using the *E. coli* strain BL 21 (DE3) and was purified using Ni-NTA and SP-Sepharose chromatography as described previously [75].

#### Terminal deoxynucleotidyl transferase–mediated dUTP nick end labeling (TUNEL) assay

To assess the apoptosis induced by HIV-1 Tat and/or morphine in human neurons, 2×10^4^ cells/well were plated in PDL coated 8-well chambered slides (Nunc, Kamstrupvej, Denmark) in complete neuronal media and treated with HIV-1 Tat and/or morphine sulphate for 24 hours with or without pre-treatment with PDGF-BB. In parallel, SHSY5Y cells were serum-starved for 2 hours before treatment with HIV-1 Tat and/or morphine sulphate for 12 hours with or without pre-treatment with PDGF-BB. To confirm whether the effects of morphine are mediated via opioid receptors, human neurons and SHSY5Y cells were treated with opioid receptor antagonist, naloxone hydrochloride dihydrate (1 µM), before the addition of morphine. After the treatments, cells were washed with phosphate buffered saline (PBS) and fixed with 4% paraformaldehyde for 20 minutes at room temperature. The fixed cells were then blocked and permeabilized with 4% bovine serum albumin containing 0.5% Triton X-100 and TUNEL assay was performed using *In Situ* Cell Death Detection Kit, TMR Red (Roche, Mannheim, Germany) as per the manufacturer's protocol. The slides were then mounted with Vectashield mounting medium containing DAPI (4,6-diamidino-2-phenylindole) (Vector Labs, Burlingame, CA, USA). Apoptosis was determined by counting the number of TUNEL-positive cells (red) and total DAPI-positive nuclei (blue) in images captured from at least five random fields using Zeiss AxioImager.Z1 microscope (Carl Zeiss, Heidenheim, Germany). For each experimental group, a minimum of 1000 cells was analyzed and apoptosis was represented as percentage TUNEL-positive cells/DAPI.

#### Reactive oxygen species measurements

To monitor the levels of oxidative stress in the cells in response to HIV-Tat and/or morphine treatment, SHSY5Y were plated at a density of 5×10^4^ cells/well in 24-well plates (Nunc, Kamstrupvej, Denmark). Cells were serum starved for 2 hours prior to treatment with HIV-Tat and/or morphine with or without pre-treatment with PDGF-BB for 30 minutes. To identify the source of free radicals, cells were treated with NADPH oxidase inhibitor, apocynin (250 µM), for 1 hour prior to addition of HIV-Tat and/or morphine. After 3 hours, cells were incubated with 5 µM DCFDA (2′, 7′-dichlorodihydrofluorescein diacetate) (Sigma Aldrich, St. Louis, MO, USA) for 1 hour at 37°C, washed twice with PBS and lysed with lysis buffer. The protein so obtained was immediately used to measure fluorescence at wavelengths of 485 nm for excitation and 530 nm for emission using Varioskan Flash Multimode Reader (Thermo Scientific, USA).

#### Analysis of mitochondrial membrane depolarization

For studying changes in mitochondrial membrane potential in human neurons, differentiated cells were plated in PDL coated 24-well plates (Nunc, Kamstrupvej, Denmark) at a density of 5×10^4^ cells/well. Cells were treated with HIV-Tat and/or morphine for 12 hours with or without pre-treatment with PDGF-BB for 30 minutes. After treatments, cells were incubated with 1 µg/ml of JC-1 (5,5′,6,6′-tetrachloro-1,1′,3,3′-tetraethylbenzimidazolylcarbocyanine Iodide) dye (Invitrogen, San Diego, CA, USA) for 10 minutes at 37°C, washed thrice with PBS and immediately visualized for JC-1 monomers (green filter) and JC-1 aggregates (red filter) using Nikon Eclipse TS100 (Nikon, Japan). For quantitative assessment of JC-1 aggregates/monomers, after incubation with JC-1 dye, cells were harvested using cold PBS, centrifuged at 350 X g, washed twice with PBS and read at 590 and 530 nm for aggregates and monomers respectively, using Varioskan Flash Multimode Reader (Thermo Scientific, USA).

#### Immunocytochemistry

After treatments, cells were fixed with 4% paraformaldehyde for 20 minutes at room temperature, washed thrice with PBS, blocked and permeabilized using 4% bovine serum albumin containing 0.2% Triton-X for 20 minutes. Human neurons/neuroblastoma cells were then incubated with anti - β 3 tubulin (1∶1000; Covance, Berkeley, CA, USA)/anti-cleaved caspase-3 (1∶200; Cell Signaling, Danvers, MA, USA) for 1 hour at room temperature/overnight at 4°C, respectively. Following washes with PBS, cells were incubated with corresponding FITC-conjugated secondary antibody (1∶200; Vector Labs, Burlingame, CA, USA) and then mounted with Vectashield mounting medium containing DAPI.

#### Western blot analysis

After treatments, proteins were isolated from SHSY5Y cells using lysis buffer [1% Triton-X 100, 10 mM Tris-HCl (pH 8.0), 150 mM NaCl, 0.5% Nonidet P (NP-40), 1 mM EDTA, 0.2% EGTA, 0.2% sodium orthovanadate and protease inhibitor cocktail (Roche, Mannheim, Germany)] and quantitated using the bicinchoninic acid (BCA, Sigma) method as described previously [74]. 30 µg of protein was electrophoresed on 10% polyacrylamide gel and then transferred onto nitrocellulose membrane (MDI, India). The membranes were blocked with 5% skimmed milk in PBS-Tween 20 (PBST) for 1 h and incubated overnight with the following antibodies: pERK1/2 (1∶2000), ERK1/2 (1∶2000), pAkt (1∶1000), Akt (1∶2000), pJNK (1∶1000), JNK (1∶1000), Bax (1∶1000), Bcl2 (1∶1000). All the antibodies were procured from Cell Signaling (Danvers, MA, USA) and prepared in 5% skimmed milk in PBST. After extensive washes with Tris buffered saline – Tween 20 (TBS-T) blots were incubated with corresponding horseradish peroxidase conjugated secondary antibodies (1∶4000, Vector Labs, Burlingame, CA, USA) for 1 hour. Blots were again washed with TBS-T and the signal was detected using chemiluminescence reagent (Millipore, Bedford, MA, USA). Images were captured using Chemi Genius Bioimaging System (Syngene, Cambridge, UK) using GeneSnap software and the images were analyzed using ImageJ software (NIH, USA). Blots were stripped and re-probed with anti-β-tubulin (1∶5000; Sigma Aldrich, St. Louis, MO, USA) as loading control.

#### Adenovirus infection

SHSY5Y cells were infected with adenoviral constructs containing the wild-type or dominant-interfering forms of Akt (kind gift from Dr. K Walsh) used at a multiplicity of infection of 50. Cells infected for 48 hrs with adenovirus constructs were subsequently treated with PDGF-BB followed by assessment of Akt phosphorylation by Western blot. Briefly, treated cells were lysed using the Mammalian Cell Lysis kit (Sigma, St. Louis, MO). Western blots were then probed with antibodies recognizing the phosphorylated forms of Akt and β-actin (Cell Signaling 1∶500). Secondary antibodies were alkaline phosphatase conjugated to goat anti mouse/rabbit IgG (1∶5000). Signals were detected by chemiluminescence (Pierce, Rockford, IL). All of the Western blot experiments were repeated three times individually and representative blots are presented in the figure 7 I.

#### Caspase-3 activity assay and immunostaining

Caspase-3 activity was analyzed using CaspACE Assay system, Colorimetric (Promega, Madison, USA) according to the manufacturer's protocol. Briefly, protein was isolated from 5×10^5^ cells/treatment group using the cell lysis buffer supplied with the kit. 100 µg of protein/treatment group was incubated in assay buffer containing 10 mM DTT and 0.2 mM caspase substrate, DEVD-pNA for 4 hours at 37°C. Post incubation, caspase-3 activity was measured using Benchmark Plus Microplate spectrophotometer (Bio-Rad Laboratories, CA, USA) at a wavelength of 405 nm. For each lysate, absorbance was normalized to the protein concentration as determined by the BCA method. For cleaved caspase-3 immunostaining, cells were processed as described earlier under the ‘Immunocytochemistry’ section. Protein kinase inhibitor, Staurosporine (Invitrogen, San Diego, CA, USA), and irreversible caspase inhibitor, Z-VAD-FMK (Promega, Madison, USA), were included in the study as positive and negative regulators of apoptosis respectively.

#### Cell viability assay

Cell viability was assessed using the 3-(4,5-Dimethylthiazol-2-yl)-2,5-diphenyltetrazolium bromide (MTT, Sigma Aldrich, St. Louis, MO, USA) assay as described previously [74]. Human neuroblastoma cells were plated in 96-well plates at a density of 1×10^4^ cells/well and serum starved for 2 hours. Specific MEK1/2 inhibitor, U0126, PI3K inhibitor, LY294002, p38 inhibitor, SB203580 or JNK inhibitor, SP600125 were added 1 hour prior to pre-treatment with PDGF-BB or treatment with HIV-Tat and morphine. After 12 hours of treatment, cells were incubated with 5 mg/ml MTT for 3 hours at 37°C. Solubilization buffer (50% dimethyl formamide, 20% sodium dodecyl sulphate) was added to dissolve any formazan crystals that formed and after 30 minutes absorbance was measured at 570 nm using Benchmark Plus Microplate spectrophotometer (Bio-Rad Laboratories, CA, USA). Values were expressed as percentage relative to those obtained in controls.

#### Statistical analysis

Experiments for each condition were performed in triplicate and repeated three to five times. Results from each set of experiment were averaged, counted as n = 1 for statistical analysis, and presented as mean ± standard deviation. Statistical significance between groups was calculated using Student's *t-test*. All values of *p<*0.05 were taken as significant.

## Supporting Information

Figure S1 **Role of MAPK pathways following exposure of human neuroblastoma neural cells with Tat and morphine.** Human neuroblastoma cells were exposed to 100 ng/ml Tat and 100 nM morphine independently as well as in combination, for the times indicated and various MAPK pathways were studied by Western blotting. (A) Densitometric analysis of ERK1/2 activation by Tat and morphine independently and in combination. (B) Morphine induced ERK1/2 activation was prevented by pre-treating SHSY5Y cells with naloxone, an opioid receptor antagonist, confirming their role in morphine induced ERK1/2 activation. (C) Densitometric analysis of phosphorylation of JNK following exposure to Tat and morphine in human neuroblastoma cells. (D) Inhibition of morphine-induced JNK activation by naloxone confirmed opioid receptor involvement. Data represents mean ± standard deviation from 3 independent experiments. * − p<0.05 as compared to untreated control; ** – p<0.005 as compared to untreated control; # − p<0.05 as compared between Tat or Morphine alone with groups as represented with solid lines over respective bars.
(TIF)
