# Supplementary figures and images for: A Growth Factor Attenuates HIV-1 Tat and Morphine Induced Damage to Human Neurons: Implication in HIV/AIDS-Drug Abuse Cases
Source: PLoS One. 2011 Mar 24;6(3):e18116. doi: 10.1371/journal.pone.0018116 (PMC3063804; doi:10.1371/journal.pone.0018116)

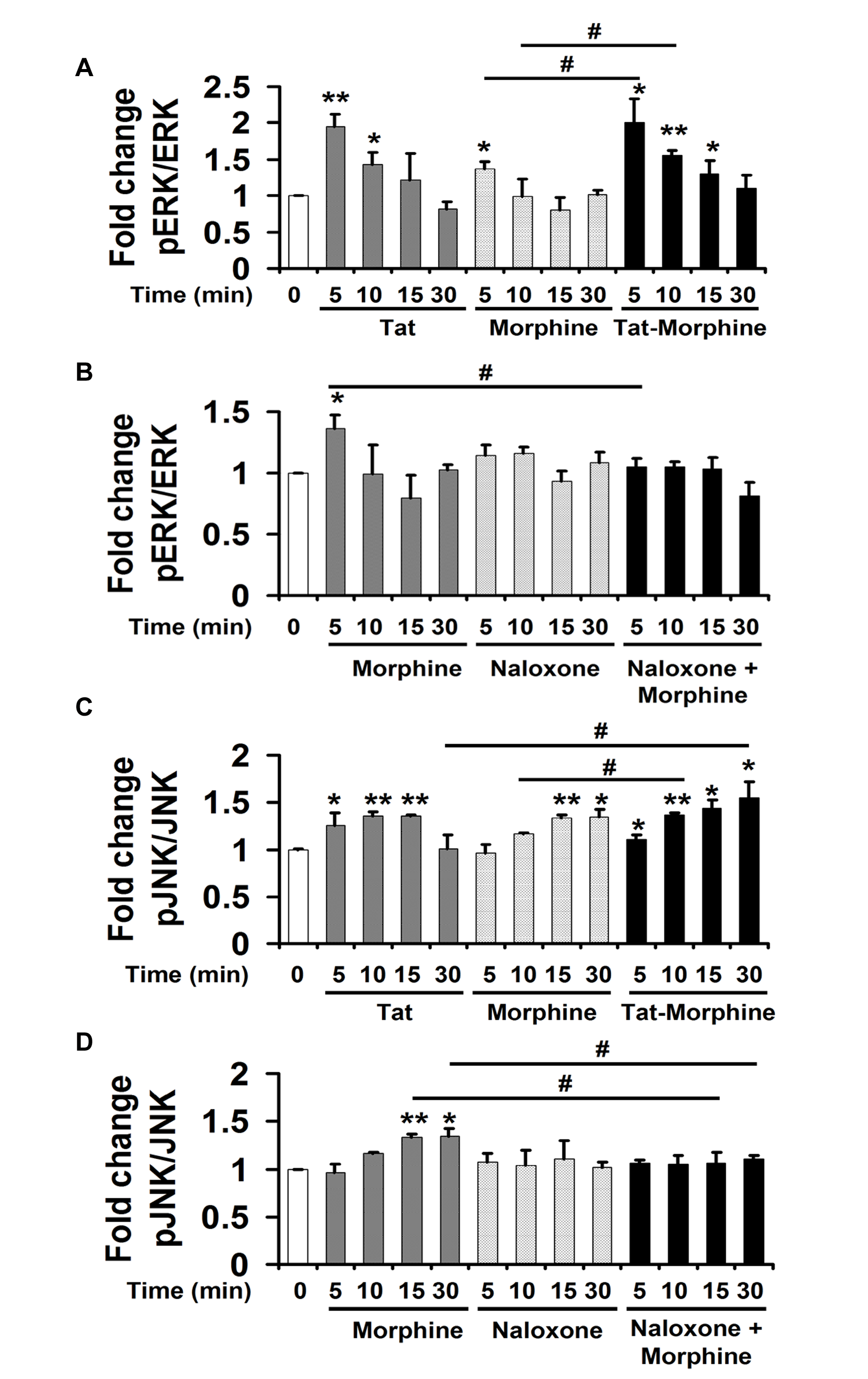

Supplement: Figure S1 — Role of MAPK pathways following exposure of human neuroblastoma neural cells with Tat and morphine. Human neuroblastoma cells were exposed to 100 ng/ml Tat and 100 nM morphine independently as well as in combination, for the times indicated and various MAPK pathways were studied by Western blotting. (A) Densitometric analysis of ERK1/2 activation by Tat and morphine independently and in combination. (B) Morphine induced ERK1/2 activation was prevented by pre-treating SHSY5Y cells with naloxone, an opioid receptor antagonist, confirming their role in morphine induced ERK1/2 activation. (C) Densitometric analysis of phosphorylation of JNK following exposure to Tat and morphine in human neuroblastoma cells. (D) Inhibition of morphine-induced JNK activation by naloxone confirmed opioid receptor involvement. Data represents mean ± standard deviation from 3 independent experiments. * − p<0.05 as compared to untreated control; ** – p<0.005 as compared to untreated control; # − p<0.05 as compared between Tat or Morphine alone with groups as represented with solid lines over respective bars. (TIF) [file pone.0018116.s001.tif]
